# Modification of Gene Duplicability during the Evolution of Protein Interaction Network

**DOI:** 10.1371/journal.pcbi.1002029

**Published:** 2011-04-07

**Authors:** Matteo D'Antonio, Francesca D. Ciccarelli

**Affiliations:** Department of Experimental Oncology, European Institute of Oncology, Milan, Italy; The Centre for Research and Technology, Hellas, Greece

## Abstract

Duplications of genes encoding highly connected and essential proteins are selected against in several species but not in human, where duplicated genes encode highly connected proteins. To understand when and how gene duplicability changed in evolution, we compare gene and network properties in four species (*Escherichia coli*, yeast, fly, and human) that are representative of the increase in evolutionary complexity, defined as progressive growth in the number of genes, cells, and cell types. We find that the origin and conservation of a gene significantly correlates with the properties of the encoded protein in the protein-protein interaction network. All four species preserve a core of singleton and central hubs that originated early in evolution, are highly conserved, and accomplish basic biological functions. Another group of hubs appeared in metazoans and duplicated in vertebrates, mostly through vertebrate-specific whole genome duplication. Such recent and duplicated hubs are frequently targets of microRNAs and show tissue-selective expression, suggesting that these are alternative mechanisms to control their dosage. Our study shows how networks modified during evolution and contributes to explaining the occurrence of somatic genetic diseases, such as cancer, in terms of network perturbations.

## Author Summary

Gene copy number is often tightly controlled because it directly affects the gene dosage. In several species, including yeast, worm, and fly, genes that have a single gene copy (singleton genes) encode proteins with several connections in the protein interaction network (hubs) as well as essential proteins. Surprisingly, in mouse and human essential proteins and hubs are encoded by genes with more than one copy in the genome (duplicated genes). Here we show that these two distinct groups of hubs were acquired at different times during the evolution of protein interaction network and contribute in different ways to the cell life. Singleton hubs are ancestral genes that are conserved from prokaryotes to vertebrates and accomplish basic functions that deal with the cell survival. Duplicated hubs were acquired mostly within metazoans and duplicated through vertebrate-specific whole genome duplication. These genes are involved in processes that are crucial for the organization of multicellularity. Although duplicated, also recent hubs are subject to gene dosage control through microRNAs and tissue-selective expression. The clarification of how the protein interaction network evolves enables us to understand the adaptation to the progressive increase in complexity and to better characterize the genes involved in diseases such as cancer.

## Introduction

Gene duplicability defines the propensity to retain multiple copies of a gene and varies among species and gene categories. In yeast, singleton genes, *i.e.* single copy genes whose duplication is selected against, preferentially encode members of protein complexes [1], highly connected [2], [3] and essential [1], [4] proteins. Similar relationships are maintained also in multicellular species such as worm and fly, where singleton genes encode highly connected [2] and essential [5] proteins. The strict retention of one single copy of these particular gene categories is a consequence of the fragility towards dosage modifications. Their duplication is deleterious because it interferes with essential cellular functions and with the fine-tuned equilibrium between formation and disruption of protein-protein interactions [6], [7].

Recent studies showed that the duplicability of mammalian hubs and essential proteins is different from that of other species. Human hubs [8], [9] and mouse essential proteins that are involved in development [5], [8], [10] are preferentially encoded by duplicated genes, while other categories of essential mouse genes can be both singletons and duplicated [5]. These differences between human, mouse and the other species suggest that gene duplicability underwent modifications during evolution, which are likely related with the extensive acquisition of novel genes in vertebrates. Through massive gene duplication followed by diversification of paralogs, vertebrates accommodated the expansion of gene families that are involved in regulation, signal transduction, protein transport, and protein modification [11], [12]. In this context, it has been proposed that a higher connectivity may favor the functional diversification of paralogs, for example through tissue specialization [8]. However, a thorough analysis of which types of genes undergo modification of their duplicability during evolution and how this influences the network properties of the encoded proteins is still missing.

The comparison of gene and network properties between species is the most straightforward approach to verify whether the modification of gene duplicability is indeed related to the expansion of the vertebrate gene repertoire. Despite the fact that current representations of protein interactomes are still incomplete [13], [14], [15] and may include a high fraction of false positives [16], the recent completion of interaction screenings in several species finally allows comparative network analyses. For example, the comparison of human, fly, worm, and yeast networks showed that they maintain a similar structure despite the difference in size [17], [18]. In addition, regardless of their connectivity, proteins that occupy central positions in the interactomes of *Saccharomyces cerevisiae*, *Drosophila melanogaster* and *Caenorhabditis elegans* are also essential and slow-evolving [18]. These studies demonstrate that the comparison of protein and gene properties in different species can be used to infer general evolutionary trends.

To unravel when the differences between duplicability and network properties arose during evolution, we undertake a comparative analysis of genes and networks in four species, *Escherichia coli*, yeast, fly, and human. These species display different levels of complexity, defined as the number of genes, cells, and cell types [11], and also high quality genomic and interaction data. We compare connectivity and centrality of all proteins with origin, conservation and duplicability of the corresponding genes. We identify a core of singleton hubs whose properties are maintained constant from prokaryotes to human, and another group of duplicated hubs that have emerged during the evolution of vertebrates. Our analysis provides evidence of how the hubs properties modified during evolution and helps in interpreting the occurrence of somatic genetic diseases that are typical of multicellularity, such as cancer, in terms of network perturbations. In particular, we find that cancer genes are representatives of the two groups of human hubs: one that originated early in evolution and is composed of singleton genes, and the other that appeared later and is enriched in duplicated genes. Functionally, these two groups correspond to *caretakers* and *gatekeepers*, suggesting that these two different ways to initiate tumorigenesis emerged at different times during evolution.

## Results

### Gene and network properties changed during evolution

**Figure 1:**
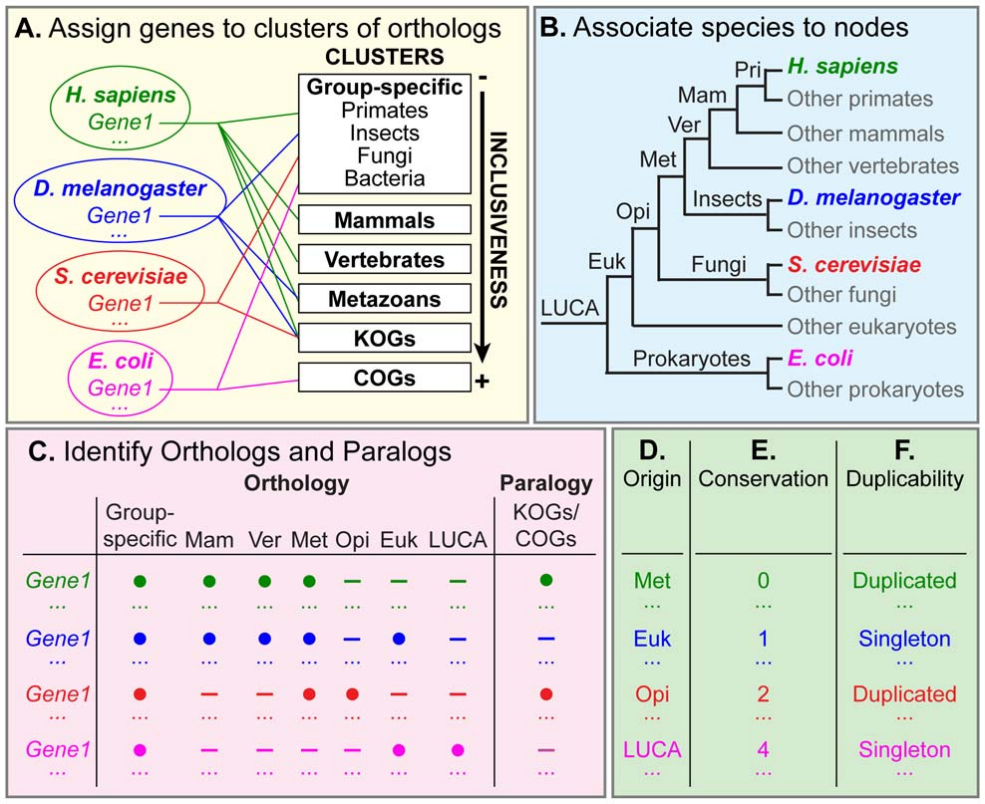
Four-step pipeline to assign gene origin, conservation, and duplicability. (A) All unique genes of the four species are assigned to clusters of orthologs with different inclusiveness. (B) All 373 species present in EggNOG [19] are associated with seven internal nodes of the tree of life. (C) Orthologs and paralogs of each gene are identified in the seven internal nodes. (D, E, F) These pieces of information are combined to identify origin, conservation, and duplicability of each gene. LUCA, last universal common ancestor.

The purpose of our analysis is to compare gene origin, conservation, and duplicability with connectivity and centrality of the encoded proteins in *E. coli*, *S. cerevisiae*, *D. melanogaster*, and *Homo sapiens*. To this aim, we identify a reliable set of unique genes in each species (Table 1), and develop a four-step procedure to determine origin, conservation, and duplicability of these genes (Figure 1). First, we retrieve all clusters of orthologs with different inclusiveness that are associated with each gene (Figure 1A) using the EggNOG database [19]. Second, we associate all 373 species present in EggNOG to seven internal nodes of the tree of life that represent major transitions in evolution (Figure 1B). These nodes include the last universal common ancestor (LUCA), which defines the ancestral organism before the split between prokaryotes and eukaryotes, eukaryotes, opisthokonts, metazoans, vertebrates, and mammals. We also consider group-specific transitions such as primates for human, insects for fly, fungi for yeast and bacteria for *E. coli*. Third, we identify orthologs and paralogs of each gene in the highest possible number of internal nodes (Figure 1C). Finally, we exploit the information collected in the first three steps to assign gene origin, conservation, and duplicability (Figure 1D, E, F).

**Table 1:** Gene sets used in the analysis.

Entries	*Hs*	*Dm*	*Sc*	*Ec*
Unique genes	22,020	13,783	6,752	4,497
Genes in EggNOG 1.0 [19]	18,205	10,543	5,411	4,196
Genes with traceable origin	18,085	10,273	5,406	4,196
Genes in KOGs/COGs	18,074	10,227	5,400	4,196
Duplicated genes (% genes in KOGs/COGs)	11,826 (65)	6,020 (59)	2,260 (42)	2,153 (51)

Protein entries present in EggNog 1.0 [19] are first associated with unique genes and then gene origin and duplicability are assigned as summarized in Figure 1 and described in the Methods. Unique entries are Entrez genes that are unambiguously associated with RefSeq v. 37 entries [54], FlyBase FB2009_01 [55], SGD (frozen at January 5^th^ 2010) [56], and EcoCyc v.14.0 [57]. *Hs*, *H. sapiens*; *Dm, D. melanogaster*; *Sc, S. cerevisiae*; *Ec, E. coli*.

**Figure 2:**
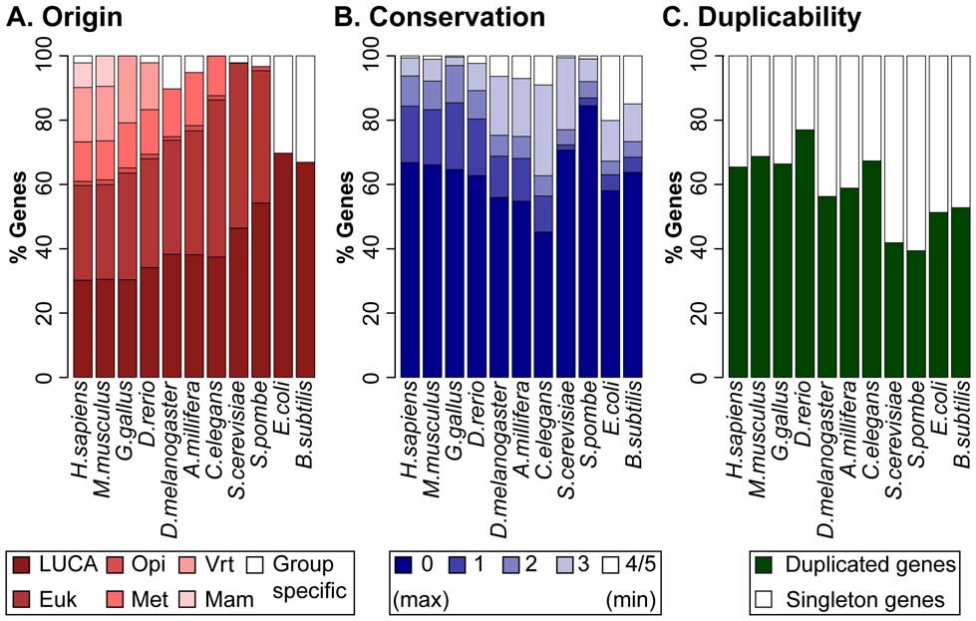
Origin, conservation, and duplicability of genes in evolution. (A) The percentage of genes that originated at each internal nodes of the tree of life is shown for the four species used in the analysis, and for seven additional species. The group-specific nodes correspond to primates for *H. sapiens*, rodents for *M. musculus*, birds for *Gallus gallus*, fishes for *D. rerio*, nematodes for *C. elegans*, insects for *D. melanogaster* and *A. mellifera*, fungi for *S. cerevisiae* and *Schizosaccharomyces pombe*, and bacteria for *E. coli* and *Bacillus subtilis*. The lack of specific genes for *C. elegans*, *G. gallus* and *M. musculus* is likely an artifact due to presence in EggNOG of few species for the corresponding group-specific nodes. LUCA, last universal common ancestor; euk, eukaryotes; opi, opisthokonts; met, metazoans; ver, vertebrates. (B) The percentage of genes that have the same conservation is shown for each species. Conservation is measured as the number of internal nodes where no ortholog is found since the gene appeared. In all species, conservation ranges from 0 (*i.e.* no missing node) to 5 (*i.e.* the gene originated with LUCA and has orthologs only in prokaryotes and in the group-specific cluster). Since only few genes have conservation 5, we grouped them with genes with conservation 4. (C) The percentage of singleton and duplicated genes is shown for all eleven species.

Since we retrieve orthologs for all species stored in EggNOG, we can use this information to infer general trends on gene origin, conservation, and duplicability during evolution. We define the evolutionary origin of a gene as the deepest internal node of the tree of life where an ortholog can be found (see Methods). Overall, we observe high variability in the gene origin between species (Figure 2A, Table S1). In accordance with previous reports [20], about 60% of human genes have orthologs in prokaryotes and early eukaryotes and more than one fourth of human genes originated with vertebrates or later. Similar trends are confirmed in other vertebrates but not in invertebrates, which are in fact composed of a higher fraction of old genes (Figure 2A, Table S1). The substantial acquisition of vertebrate-specific genes is likely related with the two events of whole genome duplications that occurred in the early vertebrate genome [21], [22].

To measure gene conservation, we count the internal nodes of the tree of life where the gene is lost since it appeared. With this measure of conservation, we do not estimate sequence divergence within a set of orthologous genes, but rather retention or loss of orthologs throughout evolution. Moreover, by counting the number of missing instead of retained nodes, we obtain estimates of conservation that are comparable between species and independent from the time of appearance of the gene. Indeed, zero always corresponds to maximum conservation, while conservation decreases progressively with the increase in the number of nodes where no orthologs can be found. Among eukaryotes, invertebrates show a lower fraction of highly conserved genes (conservation 0, 1, 2) and a higher fraction of poorly conserved genes (conservation 4 and 5) when compared to vertebrates and fungi (Figure 2B, Table S1). Coupled with the results of Figure 2A, this suggests that invertebrates retain a high fraction of ancient genes that are lost in other lineages.

To identify duplicated and singleton genes, we check whether paralogs are present within the eukaryotic-specific clusters of orthologs (KOGs) for eukaryotes, and within the most inclusive clusters of orthologs (COGs) for prokaryotes. As expected [4], gene duplicability increases with the increase in organismal complexity (Figure 2C, Tables 1 and S1). Around 65% of human genes are duplicated, and similar percentages are found in other metazoans with the exception of insects, which have less than 60% of duplicated genes, (Figure 1C, Table S1). This result, together with the high rate of DNA loss [23] and the low rate of fixed transposable elements [24], confirms the compactness of the fly genome [25].

We rebuild the interactomes of the four species by combining all available primary interaction data from seven public resources (see Methods). Given the poor overlap between these datasets, their integration considerably increases the total number of interactions (Table S2), and the resulting networks are the most complete, to our knowledge, representations of protein interactomes (Table 2). Since these resources also contain interaction data for other species, we rebuild the interactomes also for *Mus musculus* and *C. elegans* in the attempt of extending the analysis to other species. However, the resulting networks represent only around 10% and 20% of the mouse and worm proteins, respectively. Due to this high level of incompleteness, we decide not to include these species in the analysis.

**Table 2:** Protein interaction networks.

Network	Features		*Hs*	*Dm*	*Sc*	*Ec*
**All**	Proteins (% total proteins)		11,988 (54)	10,563 (77)	5,937 (88)	2,884 (64)
	Interactions		68,498	61,014	91,541	15,888
	High-Throughput (% total interactions)		29,023 (42)	58,921 (97)	77,615 (85)	15,078 (95)
	Single-Gene Experiments (% total interactions)		39,475 (58)	2,093 (3)	13,926 (15)	810 (5)
	Degree	Median	5	5	15	5
		Mean	11.4	11.5	30.9	11.0
	Betweenness	Median	898	1,011	930	287
		Mean	16,885	16,888	6,014	3,222
**Gold Set**	Proteins (% total proteins)		9,127 (42)	1,392 (10)	3,921 (58)	703 (16)
	Interactions (% total interactions)		39,868 (58)	2,236 (4)	21,721 (24)	1,004 (6)
	Degree	Median	4	2	5.5	2
		Mean	8.7	3.2	11.1	2.8
	Betweenness	Median	682	0	932	0
		Mean	14,208	2,633	6,107	618

All proteins that have at least one interaction in one of the seven original databases are reported (see Methods). The gold sets only include interactions derived from single-gene experiments or found in more than one high-throughput screening. The percentage of proteins with network information is calculated over the total unique genes for each species as reported in Table 1 and returns a rough indication of the completeness of the network. *Hs*, *H. sapiens*; *Dm, D. melanogaster*; *Sc, S. cerevisiae*; *Ec, E. coli*.

The networks of human, fly, yeast, and *E. coli* are all scale-free (Figure S1), although they differ in terms of completeness, number of interactions, and type of experimental support (Tables 2 and S2). Because of this heterogeneity, and to minimize the impact of false positives, we identify a ‘gold set’ of interactions that are supported either by single-gene experiments or by more than one high-throughput screening. The only networks that retain a substantial fraction of information are those of human and yeast (Table 2). We use these two gold sets to confirm the signal obtained from the analysis of the whole networks, thus excluding that it is affected by the experimental differences between species.

Since the networks that we rebuild are considerably bigger than those used in previous studies, as a first analysis we check whether we observe the same relationships between duplicability and connectivity that have been reported in the literature. We verify that, overall, more connected and more central proteins are encoded by duplicated genes in human and by singleton genes in the other species, both in the whole networks and in the gold sets (Figure S2). Singleton proteins are more connected than duplicated proteins also in fly, thus suggesting that the modification of the relationships between duplicability and connectivity occurred after the divergence of vertebrates.

### Ancient and conserved genes encode central hubs in all species

**Figure 3:**
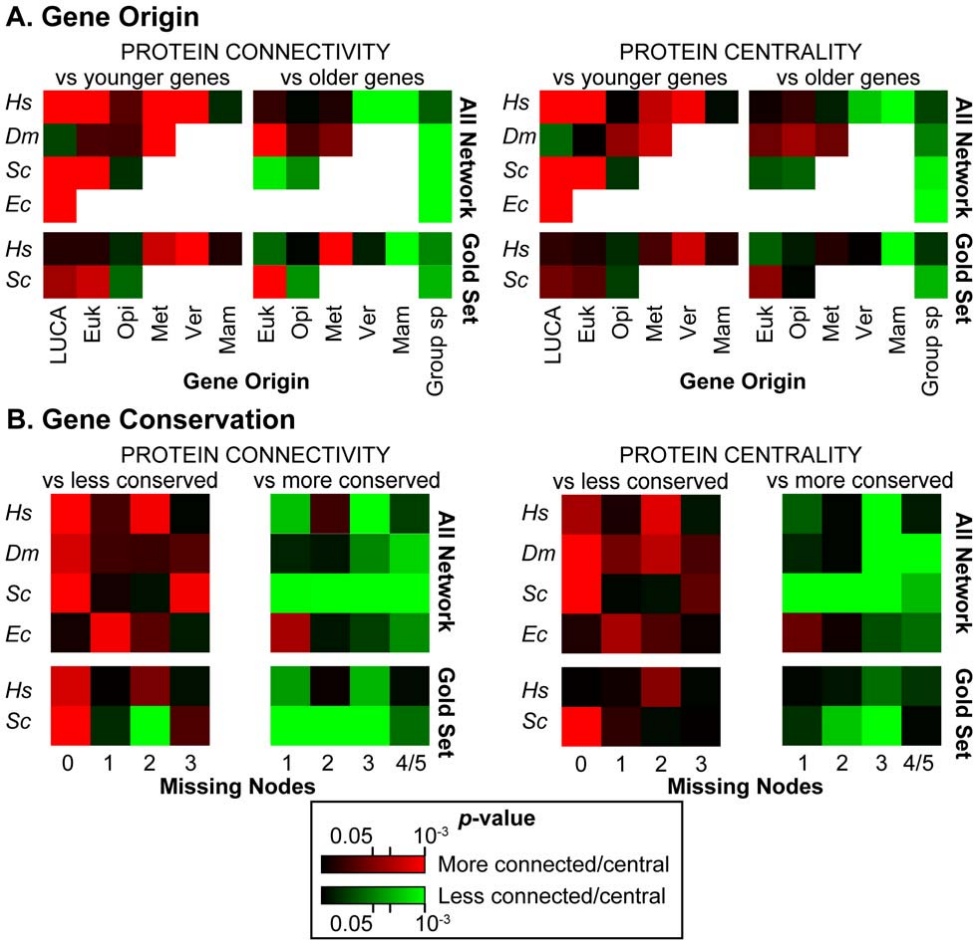
Relationship between gene origin and conservation and network properties. Degree (connectivity) and betweenness (centrality) are compared between (A) proteins that originated at a given node and younger or older proteins; and (B) proteins with a given conservation and less or more conserved proteins. In both analyses, the differences are assessed with the Wilcoxon test and the resulting *p*-values are transformed into heatmaps. Each square represents genes that originated at a given internal node or with a given level of conservation. The color represents the *p*-value. Red is associated with more connected or more central proteins, green is associated with less connected or less central proteins. The lower bound of *p*-values is set equal to 10^−3^.

In order to verify whether the time of origin of a gene affects the network properties of the encoded protein, we analyze connectivity and centrality of each protein in respect to the origin of the corresponding gene. For each species separately, we compare degree and betweenness of proteins that originated at a given evolutionary time with degree and betweenness of all proteins that originated earlier and later. In each species, we find that genes of a given age encode proteins that are significantly more connected and more central than younger proteins and less connected and less central than older proteins (Figure 3A, Table S3). This means that older proteins established more interactions and became more central during evolution. The general tendency is detectable in all four species and in the gold sets of human and yeast. The only exceptions are ancient fly genes and human genes that originated with metazoans. In fly, the unstable signal may be influenced by the high fraction of interactions detected via high-throughput experiments (Table S2), which are enriched in false positives. The higher connectivity of human proteins that originated in metazoans is instead due to the peculiar features of these genes, which will become more evident with the analysis of duplicability (see below). Since there is high variability in the number of genes that originated at each evolutionary time, we check whether this could affect the results. To this aim, we compare connectivity and centrality between random sets of 500 proteins originated at a given time and random sets of 500 younger and older proteins. After repeating the random comparison 100,000 times, we derive the distributions of the differences of mean degree and betweenness and compute the corresponding *z*-score. This is defined as the fraction of random comparisons with a difference <0 and >0 when compared with younger and older proteins, respectively. The analysis of these distributions confirms that proteins with a given origin are generally more connected and more central than younger proteins and less connected and central than older proteins (Figure S3A and Table S3).

We next verify whether also the conservation of a gene has an impact on the network properties of the encoded protein. We compare degree and betweenness of proteins with a given conservation with degree and betweenness of more and less conserved proteins. By comparing both the total distribution of degree and betweenness with the Wilcoxon test (Figures 3B) and random sets composed of an equal number of genes (Figure S3B), we observe that conserved proteins are connected and central, while proteins with low degree and low betweenness are also poorly conserved in all species. Although with a lower statistical support, the general trend is overall confirmed also in the gold sets of human and yeast (Table S3).

Our analyses show that genes that appeared early in evolution and that are well conserved encode highly connected and central proteins. Since the same trend is found independently in all four networks, it is likely that these genes constitute a core of ancestral and conserved orthologs, which maintain identical properties throughout evolution. Indeed we find that between 44 and 51% of singleton hubs that originated early in evolution in one of the four species have orthologs that are singleton hubs also in one of the other networks (Table S4). This is a remarkable result, considering the level of incompleteness of the four interactomes and the fact that they are assembled independently from each other.

### Human network acquired a novel group of duplicated hubs

**Figure 4:**
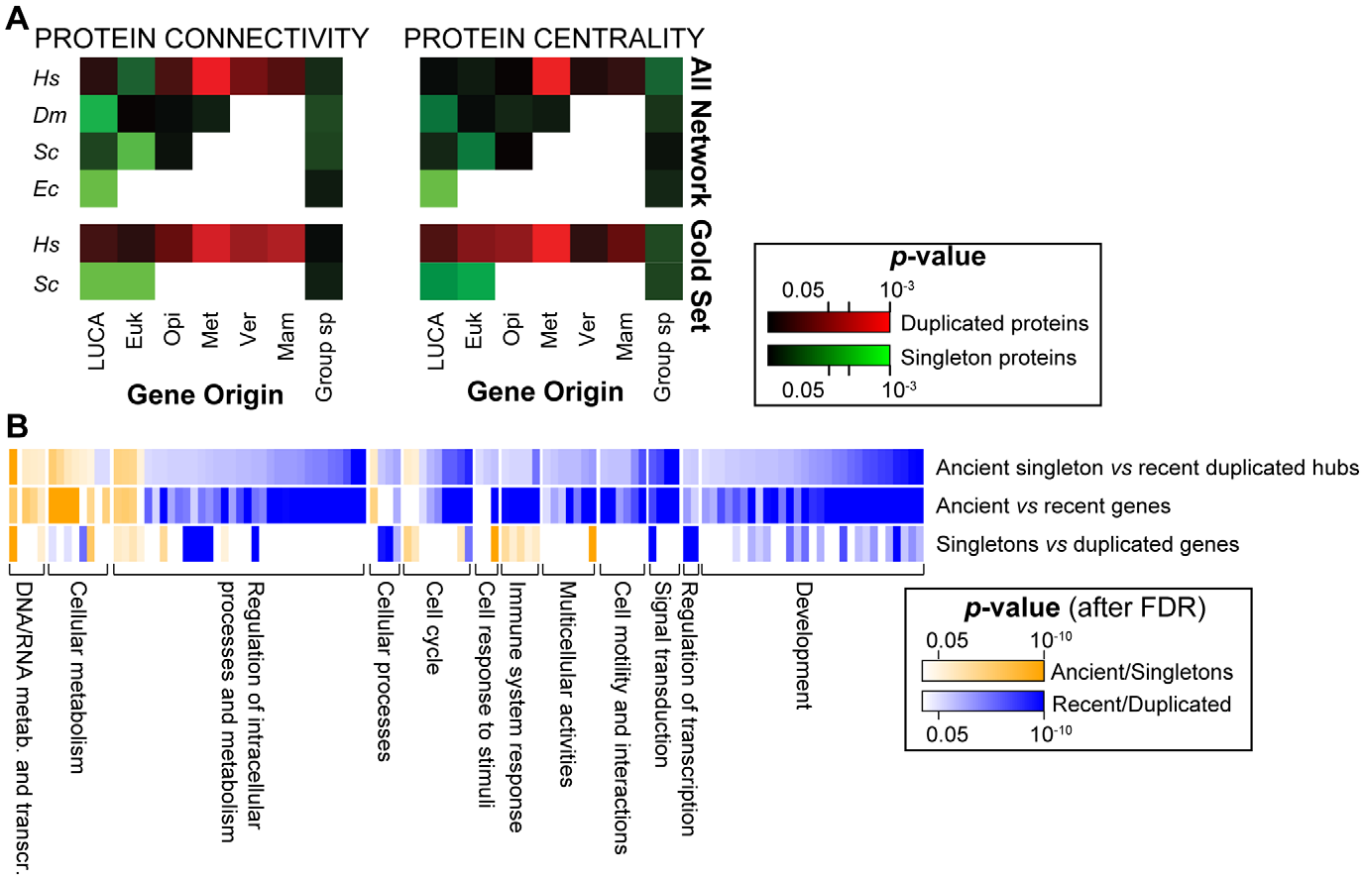
Properties of ancient and recent hubs. (A) Degree (connectivity) and betweenness (centrality) of proteins encoded by duplicated and singleton genes of same age are compared using the Wilcoxon test and the obtained *p*-values are transformed into heatmaps. Each square represents genes that originated at a given internal node and the color represents the *p*-value. Red indicates that duplicated genes encode significantly more connected or more central proteins than singleton proteins; green indicates that proteins encoded by singleton genes are significantly more connected or more central than duplicated proteins. The lower bound of *p*-values is set equal to 10^−3^. (B) Functional differences are analyzed between (1) ancestral and recent human hubs; (2) all ancestral and all recent human genes; (3) all singletons and all duplicated human genes. For each comparison, significance is assessed with Fisher's exact test and the *p*-values are adjusted for the False Discovery Rate (FDR). Vertical bars correspond to individual GO terms that are further grouped into 12 functional categories. Blue bars represent the enrichment of duplicated, recent genes, or hubs, orange represents the enrichment of singletons, ancient genes, or hubs.

Since we find that connectivity and centrality of a protein depend on when the corresponding gene appeared in evolution, we wonder how the gene origin affects the network properties of singleton and duplicated proteins. We compare connectivity and centrality between singleton and duplicated proteins that originated at the same evolutionary time. We find that, among ancient genes (*i.e.* genes originated with LUCA and in early eukaryotes), singletons encode more connected and more central proteins than duplicated genes (Figure 4A, Table S5). Surprisingly, this tendency is detectable in all four species, including human, despite the opposite general trend of the human network (Figure S2). The difference between human and the other species arises when younger genes are analyzed. Human duplicated genes that originated with metazoans encode more connected and more central proteins than singleton genes of comparable age (Figure 4A). For connectivity, this tendency is detectable also for genes that appeared in vertebrates and in mammals, although with lower statistical support. Again, the trend is confirmed in the gold sets (Figure 4A).

According to our findings, all species from prokaryotes to vertebrates maintain a group of highly connected proteins, which are encoded by ancient, conserved, and singleton genes that are sensitive to dosage modification. Another group of human hubs emerged later in evolution, namely with metazoans and, to a lower extent, with vertebrates and mammals. These genes differ from ancient hubs because they can retain gene duplicates and are therefore robust towards gene duplication. Their high connectivity explains why human genes that originated in metazoans deviate from the common trend and are more connected and central than older genes (Figures 3 and S3). In fly, the network properties of duplicated proteins that originated with metazoans do not differ from those of singletons. Therefore, metazoan-specific genes became central hubs at least after speciation of insects. This once again confirms that the modification in the relationships between duplicability and connectivity occurred in the ancestor of vertebrates.

### Ancient and recent human hubs accomplish different functions

According to the results of our analysis, human hubs can be divided into two groups depending on their origin and duplicability. To test whether this distinction also results in the accomplishment of different biological processes, we compare the functions of these two groups of hubs. In absence of a consensus definition [26], we identify hubs as the top 25% most connected proteins of the network, This results in 2,573 human proteins with more than 12 interactions. The comparison between the two groups of hubs shows that they are indeed involved in different processes (Figure 4B, Table S6). Ancient singleton hubs are enriched in basic functions that are needed for the survival of the cell, such as cellular metabolism and transcription. Duplicated hubs that appeared recently in evolution are instead involved in regulatory functions that coordinate the organization of the multicellular organism (Figure 4B). We also notice that the time of appearance of a gene affects its function more than the duplicability (Figure 4B, Table S6). Ancient and recent hubs are therefore representative subgroups of ancient and recent genes, respectively. Similar functional differences between ancient and recent genes have been reported in yeast, where ancestral genes are involved in transcription, replication, and other basic cellular processes, while genetic, transcriptional, and posttranslational regulation is associated with recently evolved genes [27].

### Gene dosage of human duplicated hubs is tightly regulated

To understand how duplicated hubs adapted to the dosage imbalance due to gene duplication, we check whether they are ohnologs, *i.e.* paralogs originated via whole genome duplication [28], miRNA targets, and tissue-selective genes. These are three different ways of controlling gene dosage. The duplication of the entire genome maintains the dosage balance between interactors and allows the duplication of dosage-sensitive genes in yeast [29] and in vertebrates [30]. Similarly, miRNAs play a pervasive role in the post-transcriptional regulation of gene expression in higher eukaryotes, particularly in those biological processes that require a fine-tuned control of the gene dosage, such as signal transduction [31]. Finally, tissue selectivity represents yet another mechanism of gene dosage control because paralogs expressed in different tissues do not interfere with each other [32], [33].

**Figure 5:**
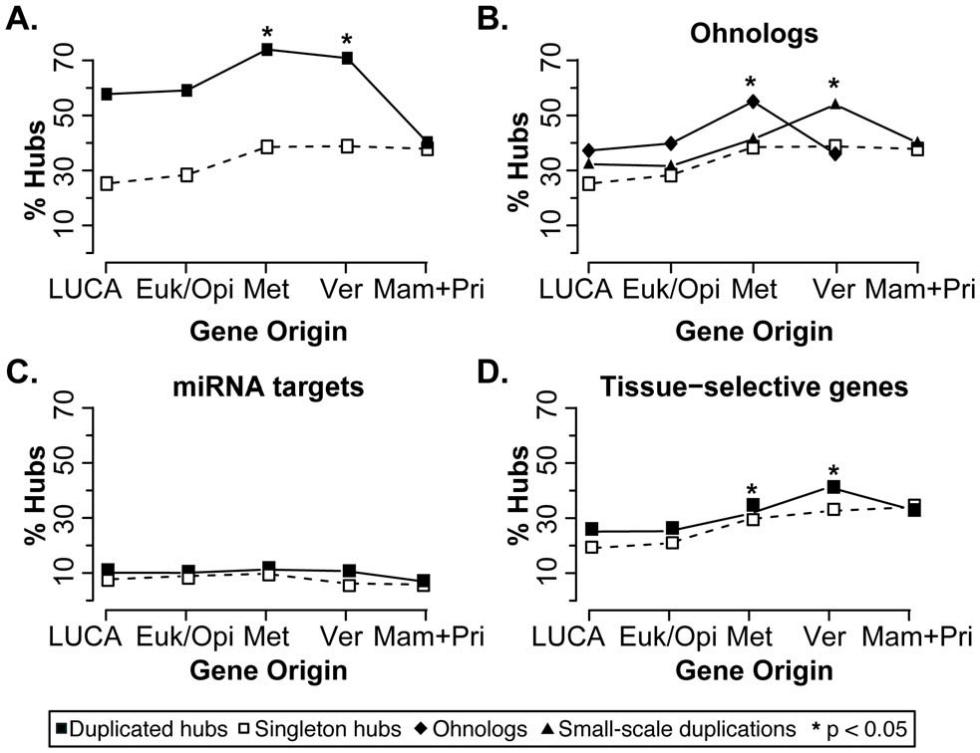
Dosage regulations of human hubs. (A) The fraction of human duplicated hubs that are ohnologs, miRNA targets, and tissue-selective genes is compared to the corresponding fraction of singleton hubs. Although the main contribution is due to ohnologs (B), the enrichment still remains detectable when miRNA targets (C) and tissue-selective genes (D) are considered separately. Small-scale duplications refer to duplicated hubs that are not the result of whole-genome duplication (*i.e.* they are not within the dataset of ohnologs). Since the number of hubs that originated with opisthokonts and primates is only 43 and 17, we group them with hubs that originated with eukaryotes and mammals, respectively. * significant enrichment when compared to older genes (Fisher's exact test).

We find that the fraction of duplicated hubs that are also ohnologs, miRNA targets, and tissue selective genes is significantly higher than that of singleton hubs (Figure 5A, 61.4% and 33.9%, respectively, *p*-value <2.2×10^−16^, Fisher's exact test). This enrichment is mostly due to the large overlap between ohnologs and duplicated hubs (Figure 5B). However, the same trend remains detectable when only miRNA targets (Figure 5C), and tissue selective genes (Figure 5D) are considered separately. Within duplicated hubs, these types of dosage regulation act on genes that appeared in metazoans and vertebrates more frequently than on genes that appeared earlier (Figure 5).

**Figure 6:**
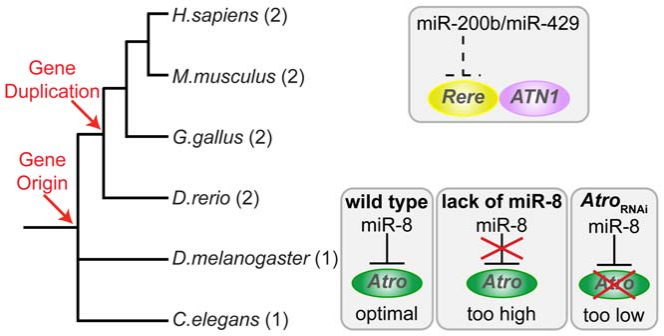
Dosage regulation of the *atrophin* genes. *Atrophins* are metazoan-specific genes that underwent duplication in vertebrates. The fly ortholog *Atro* is highly dosage sensitive: increased and reduced expression due to modifications of miR-8 lead to neurogenerative and survival defects [38], [39]. *Rere*, one of the two vertebrate atrophin paralogs, is target of mir200b and miR-429, the vertebrate counterparts of miR-8. Dosage modifications of *Rere* lead to re-localization of the other paralog, ATN1, in the nucleus, upon direct binding [41]. Interestingly, *ATN1* is the gene responsible for the dentatorubral-pallidoluysian atrophy (DRPLA) [40].

One example that explains the role of miRNAs in tuning the gene dosage of paralogs is represented by atrophins, a phylogenetically conserved family of transcriptional regulators that appeared in metazoans (*Atro*) and duplicated in vertebrates (*ATN1* and *Rere*, Figure 6). *Atrophins* are broadly expressed particularly during development [34], [35], [36], and their modification leads to neurodegenerative defects in fly [36] and in vertebrates [37]. The dosage of the fly atrophin gene *Atro* is under the tight control of the microRNA miR-8 [38] (Figure 6). The lack of miR-8 produces *Atro* overexpression and results in elevated apoptosis in the brain, behavioral defects and severe defects in animal survival [38], [39]. Also reduced *Atro* expression causes impaired survival, indicating that the fine-tuning dosage of this gene is crucial for its activity [38]. The gene dosage balance of the two *atrophin* paralogs seems to be tightly regulated also in vertebrates. Indeed, the Rere protein is able to directly bind the other atrophin paralog ATN1, which is responsible for the neurodegenerative disorder dentatorubral-pallidoluysian atrophy (DRPLA) [40], and to induce its massive re-localization in the nucleus upon overexpression [41]. Due to this direct interaction, it has been speculated that the modifications of *Rere* gene dosage may have a role in the pathogenesis of DRPLA [42]. Interestingly, *Rere*, but not *ATN1*, is the target for the counterparts of miR-8, *i.e.* miR-200b and miR-429 (Figure 6), which may regulate its dosage in a similar way [38]. In this scenario, it is reasonable to support a possible role of miR-200b and miR-429 in regulating the dosage balance between the two vertebrate *atrophin* paralogs.

## Discussion

In this study we show that the evolutionary history of a gene affects its duplicability, as well as the centrality and the connectivity of the encoded protein in the corresponding interactome. These results offer novel insights into the reciprocal influences between gene and network modifications during evolution. In all species, the core of the network is composed of ancestral and singleton hubs that are highly conserved because they do not require further modifications. Genes that are progressively acquired during evolution instead encode less connected and less central proteins. This agrees with the observation that essential proteins occupy the center of the network [13], while proteins that are under positive selection and undergo structural modifications are located at the network periphery [43]. The importance of the time of origin on the properties of a gene has been recently reported also in yeast where proteins that originated before the whole genome duplication are more connected and more central than younger proteins [44]. Intuitively, these results support the preferential attachment model of network evolution, in which the expansion of the network starts from an ancient core [45] and progresses through gene duplication and divergence [46]. However, our analysis also reveals that significant deviations from this model occur in correspondence of massive genome reorganizations, such as the whole genome duplications that occurred in vertebrates. Owing to such events, even genes that are sensitive to dosage modifications can tolerate duplications because the dosage balance with their interactors is preserved. Therefore, together with the increase in the number of protein coding genes, vertebrates also modified their interactomes and likely both events played a role in shaping their evolution.

The rapid functional divergence of paralogs through massive neo- and sub-functionalization [47], [48] could also explain the retention of paralogous hubs owing to the quickly diversification of their function. However, sub- and neo-functionalization play a role in the diversification of paralogs also in other species such as *E. coli*, yeast, and fly, where only singleton hubs are retained. Therefore, the time of origin, more than the functional divergence, influences the retention of duplicated hubs.

Conceptually and functionally, the two evolutionary distinct groups of ancient and recent human hubs resemble ‘date’ and ‘party’ hubs that have been described in the yeast interactome [49]. Similarly to party hubs, ancestral and singleton human hubs are mainly involved in cellular and nucleic acid metabolism, while recent and duplicated human hubs act as regulators, mediators or adaptors, similarly to date hubs. The difference between yeast and human is again in the time of appearance of human duplicated hubs and in the fact that in yeast both groups are encoded by singleton genes. Moreover, in human the signal of high connectivity and centrality that derives from recent hubs is stronger than that from ancient hubs (Figures 3, S3, 4 and Table S5). This is consistent with previous findings of an overall enrichment of the human network in duplicated hubs [8], [9] (Figure S2).

There are several indications that, despite being robust towards gene duplication, recent hubs remain sensitive to gene dosage modifications. First, human duplicated hubs rapidly underwent alternative ways to control their dosage, for example through tissue-selective expression and miRNA regulation (Figure 5). Second, ohnologs do not undergo further small-scale duplications and copy number variations [30]. Finally, genes that carry disease-related germline mutations are depleted in hubs [50] and somatic mutations of hubs are often associated with cancer [9], [51], [52]. All together, these observations indicate that hub modifications are usually harmful, even independently from the individual gene function. This analysis also adds novel insights to our understanding of the network properties of cancer genes and to the importance of gene dosage in the development of cancer. We recently reported that cancer genes are overall enriched in singleton hubs [9]. However, when the same analysis is repeated taking into account the gene origin, also cancer genes, like other human hubs, can be divided into two groups (Figure S4). One group is composed of ancestral cancer genes that encode singleton hubs, while the other includes cancer genes that originated with metazoans and are enriched in duplicated hubs. These two groups of cancer genes broadly correspond to *caretakers*, *i.e.* genes involved in the repair of DNA and in the maintenance of genome stability, and *gatekeepers*, which instead appeared lately in evolution and accomplish functions related to signaling and growth [53]. Therefore, there are two ways of promoting cancer, one that deals with basic and ancestral functions, and the other that interferes with regulatory processes. In either case, tumorigenesis starts from the somatic perturbation of hubs, which represent components of the cellular network that are sensitive to modifications.

## Methods

### Gene sets and reconstruction of protein interaction networks

For the four species considered in the analysis (*H. sapiens*, *D. melanogaster*, *S. cerevisiae* and *E. coli*), we only use the protein entries present in EggNOG v. 1.0 [19] that are associated with unique gene identifiers. As sources of unique genes we consider RefSeq v. 37 entries [54] for human; FlyBase FB2009_01 [55] for fly; SGD (frozen at January 5^th^ 2010) [56] for yeast; and EcoCyc v.14.0 [57] for *E. coli*.

We gather protein-protein interactions from the non-redundant integration of seven public resources: BioGRID v. 2.0.49 (February 1^st^ 2009) [58], IntAct (frozen at January 23^rd^ 2009) [59], MINT (frozen at February 5^th^ 2009) [60], DIP (frozen at January 26^th^ 2009) [61], DroID v. 4.0 (July 2008) [62], HPRD (September 1^st^ 2007) [63], and a recent map of yeast interactions detected by yeast-two-hybrid [16]. We only consider primary data (*i.e.* interactions directly detected in each of the species), and discard putative interactions inferred from orthology. We distinguish between two types of experimental evidence: 1) single-gene experiments, *i.e.* studies that report less than 100 interactions; and 2) high-throughput experiments associated with large-scale screenings. We derive a gold set of interactions that only includes data that are supported by single-gene experiments or by more than one high-throughput screening. For each protein in the four networks we compute degree and betweenness. Degree measures the connectivity of a protein inside the network and is calculated as the number of binary interactions. Betweenness is a measure of centrality and is related to the number of shortest paths that pass through a protein [64].

### Orthology and paralogy assignment

We identify seven internal nodes of the tree of life that correspond to major transitions in evolution (LUCA, eukaryotes, opisthokonts, metazoans, vertebrates, mammals, and group-specific transition), and assign each of the 373 species present in EggNOG v. 1.0 to the most specific internal node, using the corresponding taxonomy ID. The four analyzed species are assigned to the corresponding group-specific transition (primates, insects, fungi, bacteria), while the remaining 369 species are taken as representatives of the other major transitions. For example, we assign human to primates, other non-primate mammalian species (*i.e.* mouse) to mammals, non-mammalian vertebrate species (*i.e.* fish) to vertebrates, and so on. The group-specific nodes for the four species do not reflect comparable evolutionary transitions, and for human we are much more specific than with the other three species. This reflects the availability of species and orthology information in EggNOG. For example, in human we are able to discriminate between genes that originated in mammals and genes that originated with primates because in EggNOG there are three primates (*H. sapiens*, *Pan troglodytes* and *Macaca mulatta*) and five additional mammals (*Monodelphis domestica*, *Bos taurus*, *Canis familiaris*, *M. musculus* and *Rattus norvegicus*). For fly, instead, the group-specific transition is insects, because only three insects have orthology information (*D. melanogaster*, *Apis mellifera* and *Anopheles gambiae*). It should be noted that this different resolution of the group-specific nodes does not introduce any bias in the results because the fraction of group-specific genes is very low in all species. In addition, the number of genes that originated at a certain time in evolution does not affect genes that originated earlier or later. Finally, the general trend of origin, conservation, duplicability and network properties is detectable in all species, independently on the resolution of the group-specific transitions.

Once species have been assigned to internal nodes, we assign each gene to clusters of orthologs with different levels of inclusiveness and check for the presence of orthologs in the seven internal nodes. For example, for human we check for the presence of non-primate orthologs in the mammalian clusters, of non-mammalian orthologs in the vertebrate clusters, of non-vertebrate orthologs in the metazoan clusters, and so on.

### Evolutionary origin, conservation, and duplicability

We define the origin of each gene as the most ancient internal node where an ortholog can be found. For a small number of genes in each species (120 in human, 270 in fly and 5 in yeast) we cannot assign a precise evolutionary origin, because no clusters that contain the gene include representative orthologs. These genes are excluded from further analysis. To measure conservation of a gene throughout evolution, we count the number of missing nodes, *i.e.* internal nodes of the tree of life where no orthologs of that gene can be found since it originated. By considering the same number of internal nodes (seven) for all species and by counting the number of lost instead of retained nodes, we gather an estimate of conservation that is comparable between species and independent from the origin of the gene. We consider a gene duplicated if there is at least one other gene of the same species (*i.e.* at least one paralog) within the eukaryotic-specific clusters (KOGs) for human, fly and yeast, and within the most inclusive clusters (COGs) for *E. coli*. If no paralogs can be detected, the gene is considered singleton. With this method, we do not date the time of gene duplication but rather gene duplicability, *i.e.* whether a gene underwent duplication and this duplication was retained at least once in evolution. For a total of 63 genes in human, fly, and yeast both KOG nor COG clusters are available, and we exclude these genes from further analysis.

### Comparison of gene and network properties

We group genes according to their evolutionary origin and compare the distributions of degree and betweenness with the corresponding distributions of younger and older proteins. In a similar way, we compare the distributions of degree and betweenness of proteins with a given conservation with those of more and less conserved proteins. All comparisons are made using the Wilcoxon test. In order to eliminate possible biases due to the different number of genes that originated at each evolutionary time, we apply a randomization test. In each species independently, we extract 500 random genes with a given origin and calculate the mean degree and betweenness of the corresponding proteins. We then compute the difference between these values and the corresponding mean degree and betweenness of 500 randomly picked younger proteins and older proteins, separately. In case a group includes less than 500 genes, also the other groups will contain the same number of genes (*i.e.* since there are only 84 primate-specific genes, they are compared to 84 randomly selected younger or older genes). We repeat the random comparison 100,000 times and derive the distributions of the degree and betweenness differences between the proteins that originated at a certain evolutionary level and younger and older proteins. Finally, we calculate the *z-*score as the fraction of random comparisons with a difference <0 when comparing with younger proteins, and >0 when comparing with older proteins. Differences in the mean degree or betweenness <0 are associated with more connected or central proteins, while differences >0 to less connected or central proteins. We use a similar random test to compare degree and betweenness of proteins with a certain level of conservation with more and less conserved proteins. To visualize the results, we transform the *p*-values and *z-*scores into heatmaps. Red boxes are associated with significantly higher values of degree and betweenness, green boxes correspond to significantly lower values, and non-significant *p*-values are colored in black. To evaluate the effect of gene origin on duplicability, we compare degree and betweenness of duplicated and singleton proteins with the same age using the Wilcoxon test. Also in this case, we derive the heatmaps from the *p*-values. Red-colored boxes indicate that duplicated proteins are more connected or more central, green-colored boxes indicate that singleton proteins are more connected or more central, and black indicates no statistically significant difference between singleton and duplicated proteins.

### Functional analysis

To perform the functional analysis we rely on the biological process branch of the gene ontology (GO) tree and compare GO terms present at levels 5 and 6 [65]. GO levels refer to the branching points of the tree, with level 1 corresponding to the root of the tree. Increase in levels numbers are associated with increased specificity in the functional description and to decreased number of described genes. Levels 5 and 6 represent a compromise to obtain a good resolution in functional description for a fair number of genes. We further group all terms at these two levels into 12 categories and perform three comparisons: (1) ancient singleton hubs and recent duplicated hubs; (2) genes that originated in LUCA and eukaryotes (ancient) and genes that originated in metazoans and vertebrates (recent); (3) singletons and duplicated genes. For each comparison, the functional enrichment is detected using Fisher's exact test and the resulting *p*-values are adjusted for the false discovery rate (FDR) using Benjamini-Hochberg method.

### Ohnologs, miRNA targets, and tissue-selective genes

From the list of 4,174 human ohnologs, *i.e.* paralogs originated via whole genome duplication [22], we identify 3,867 genes in our dataset that duplicated through whole genome duplications. Of these, 3,618 are duplicated genes, while the remaining 249 singletons are likely false positives and thus discarded from further analysis. To derive a list of human genes that are targets of microRNAs, we use Tarbase v.5 (June 2008) [66] and miRecords v.1 (August 15, 2008) [67], which collect 1,051 and 1,311 experimental interactions, respectively. Starting from the interactions, we derive 986 human miRNA target genes from the two lists (Table S7). Of these, 952 genes are also present in our dataset of 18,074 unique human genes. We retrieve expression data for 13,787 unique Entrez genes in 36 [68] and in 73 [69] human normal tissues (six tumoral tissues were excluded from the analysis to avoid that the deregulation of gene expression due to the disease condition could influence the analysis). We obtain a cumulative dataset of 4,988 tissue-selective genes, by considering only genes that are expressed in less than 25% of the analyzed tissues (8 and 17 in the two studies, respectively). Of these, 4,616 genes are also present in our list (Table S8).

From the obtained lists of ohnologs, miRNA targets and tissue-selective genes, we extract the genes that encode duplicated and singleton hubs (Table S9). We then compare the corresponding fractions of singleton and duplicated hubs that are also ohnologs, miRNA targets and tissue-selective genes altogether and separately.

All statistics are done using R version 2.10.1.

## Supporting Information

Figure S1 Degree distribution of protein interaction networks in the four species. The degree represents the number of interactions of each node in the network, while *P* represents the probability of a node to have a certain degree. The blue line indicates the power-law interpolated from the nodes with degree >10. The exponent gamma ranges between 2.09±0.04 for yeast and 2.21±0.04 for fly, so all the four networks can be considered scale-free [45], [70]. In order to determine whether the calculated power-law adequately fits the degree distributions, we use Kolmogorov-Smirnov test, with the null hypothesis that the power-law line fits the data. Since the *p*-values from the Kolmogorov-Smirnov tests are all not significant, the null hypothesis cannot be rejected and the calculated power-law is an adequate descriptor of the degree distributions for all four networks. C.I., confidence interval.
(TIF)

Figure S2 Connectivity and centrality of singleton and duplicated genes in the four networks. Degree and betweenness of proteins encoded by all duplicated and all singleton genes are compared in the four species using the Wilcoxon test. All *p*-values are transformed into heatmaps where red indicates that duplicated genes encode for significantly more connected or more central proteins than singleton proteins. Green indicates that proteins encoded by singleton genes are significantly more connected or more central than duplicated proteins. Black indicates non-significant *p*-values. This analysis is done using the entire network for all the four species and the gold set for human and yeast.
(TIF)

Figure S3 Relationship between gene and network properties measured with randomization tests. Degree (connectivity) and betweenness (centrality) are compared between (A) proteins that originated at a given node and younger or older proteins; and (B) proteins with a given conservation and less or more conserved proteins. In each species, we pick subsets of 500 random genes with a given origin, determine the mean degree and betweenness of the corresponding proteins and compute the difference with 500 younger and 500 older proteins. We repeat the same procedure 100,000 times and derive a *z-*score as the fraction of randomizations with a negative difference when comparing with younger proteins, and with a positive difference when comparing with older proteins. The same analysis is done for conservations. Each square in the heatmap represents genes that originated at a given internal node or with a given level of conservation. The color represents the *z*-score. Red is associated with more connected or more central proteins, green is associated with less connected or less central proteins. The lower bound of *z*-scores is set equal to 10^−3^.
(TIF)

Figure S4 Time of appearance of recessive and dominant cancer genes. The percentage of genes that originated at each of the seven internal nodes of the tree of life is compared between cancer genes and the rest of human genes. The 415 cancer genes are derived from the cancer gene census (frozen at January 11^th^ 2010), and are defined as genes that are causally implicated in tumorigenesis [71]. For 393 of those, the origin can be traced (Table 1), and 310 genes are defined as dominant, and 85 as recessive. Two genes (*CBL* and *PKRAR1A*) are included in both lists because they can behave as dominant and recessive. Differences between the appearance of cancer genes and the rest of human genes are calculated using Fisher's exact test and, where significant, are depicted as diagonal lines.
(TIF)

Table S1 Origin, conservation and duplicability of genes in evolution. For each species, origin (A), conservation (B) and duplicability (C) are assigned as summarized in Figure 1 and described in the Methods. The total genes correspond to the genes present in KOGs/COGs, as reported in Table 1. Conservation ranges from 0 (i.e. no missing node) to 5 (i.e. the gene originated with LUCA and has orthologs only in prokaryotes and in the group-specific cluster). Since only few genes have conservation 5, we grouped them with genes with conservation 4.
(XLS)

Table S2 Source of the protein-protein interaction data used in the analysis. The number of proteins, interactions, and experiments (counted as number of Pubmed IDs that support each interaction) are reported for each species in each database.
(XLS)

Table S3 Connectivity, centrality, time of origin and conservation of genes in the four species. (A) Network properties of genes that originated at each time in evolution are compared with those of genes that originated earlier and later in evolution. The distribution of degree and betweenness of younger and older genes are compared using the Wilcoxon Test and with 100,000 randomizations of subsets of 500 proteins in order to eliminate eventual biases due to the comparison of groups with different numbers of genes. *P*-values and *Z*-scores <0.05 are reported in bold (green for depletion, red for enrichment). NA  =  not available. (B) Network properties of genes that have a certain level of conservation are compared with more and less conserved genes. Conservation is calculated on the basis of the number of internal nodes where no orthologs of each gene are found. The distribution of degree and betweenness of more and less conserved genes are compared using the Wilcoxon Test and with 100,000 randomizations of subsets of 500 proteins, as in (A).
(XLS)

Table S4 Orthology relationship between ancient singleton hubs. In each species, the orthologs of singleton hubs that originated early in evolution (LUCA and Eukaryotes) and with network information in at least one of the other model species are extracted. Then the number of hubs that have at least one ortholog that is also hub in its protein interaction network is calculated.
(XLS)

Table S5 Connectivity and centrality of singleton and duplicated genes. Connectivity is measured using the degree, while centrality using the betweenness of each node in the networks. The distribution of degree and betweenness between singleton and duplicated genes are compared using the Wilcoxon Test. *P*-values <0.05 are reported in bold.
(XLS)

Table S6 Functional analysis. Three functional comparisons are performed on the basis of the terms at levels 5 and 6 of the biological process branch of GO: (A) Comparison between recent duplicated hubs and ancestral singleton hubs, (B) recent and ancestral genes, and (C) duplicated and singleton genes. Recent genes originated with metazoans or vertebrates; ancestral genes originated with LUCA or eukaryotes.
(XLS)

Table S7 Human miRNA targets. For each of the 986 human genes that are targets of miRNAs, the original source, type of experimental support and corresponding Pubmed ID(s) are displayed. SG, single-gene experiment; MA, microarray, MS, mass-spectrometry. 1 represents presence of the gene in the dataset or experimental data, 0 represents absence.
(XLS)

Table S8 Gene expression data. The number of tissues includes all non-cancer tissues from the original analyses by Ge [68] and Su [69]. The intersection with the 18,074 genes that have origin and duplicability information is also indicated.
(XLS)

Table S9 Dosage regulation of human hubs. For each evolutionary time point, the fraction of singleton and duplicated hubs that are also ohnologs, miRNA targets or encoded by tissue-selective genes is compared. Opisthokonts are grouped with eukaryotes and primates with mammals because the number of hubs that originated with opisthokonts and primates is too low to make any statistical analysis. * total duplicated hubs; ** total singleton hubs.
(XLS)
