# Supplementary figures and images for: Modification of Gene Duplicability during the Evolution of Protein Interaction Network
Source: PLoS Comput Biol. 2011 Apr 7;7(4):e1002029. doi: 10.1371/journal.pcbi.1002029 (PMC3072358; doi:10.1371/journal.pcbi.1002029)

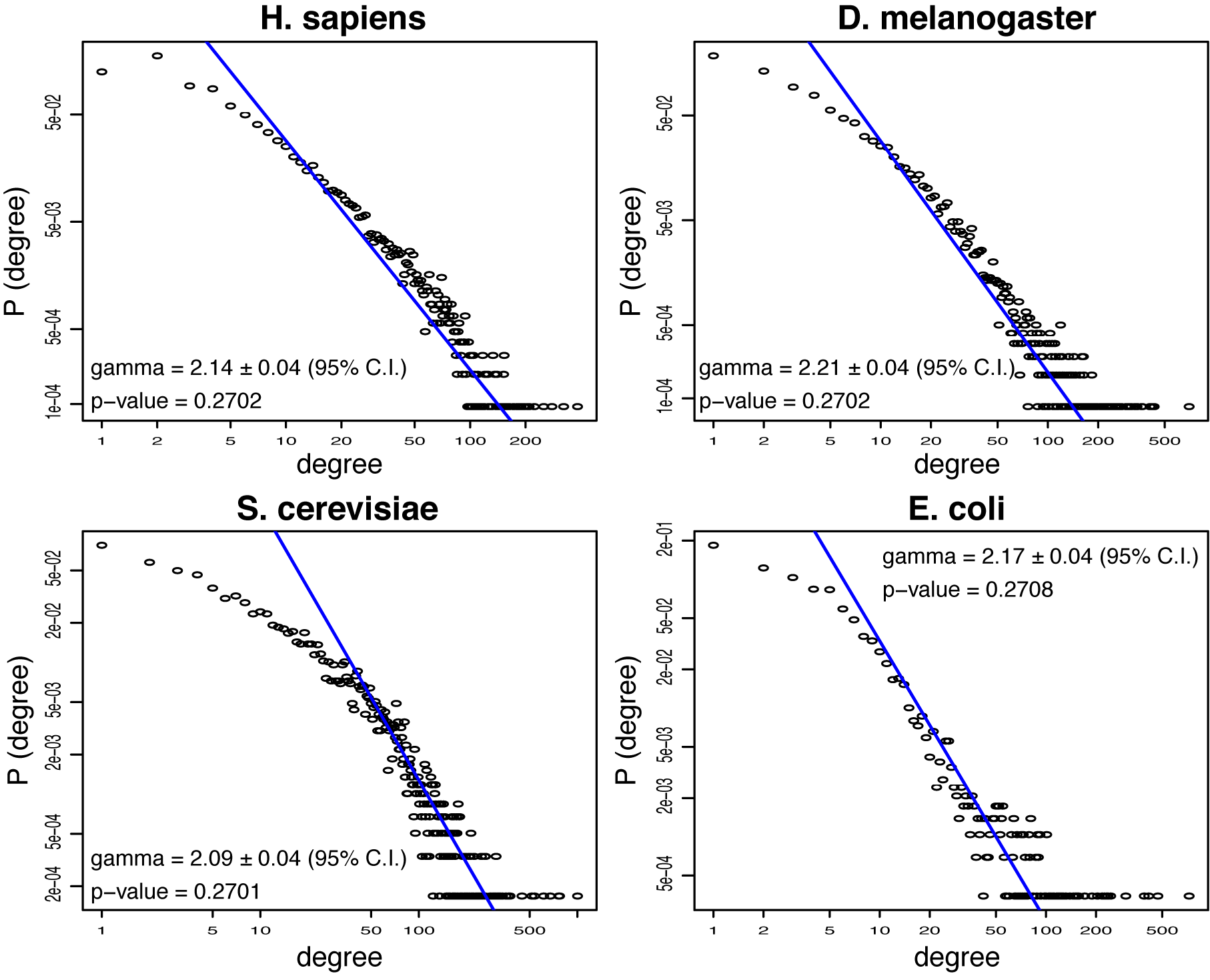

Supplement: Figure S1 — Degree distribution of protein interaction networks in the four species. The degree represents the number of interactions of each node in the network, while P represents the probability of a node to have a certain degree. The blue line indicates the power-law interpolated from the nodes with degree >10. The exponent gamma ranges between 2.09±0.04 for yeast and 2.21±0.04 for fly, so all the four networks can be considered scale-free [45], [70]. In order to determine whether the calculated power-law adequately fits the degree distributions, we use Kolmogorov-Smirnov test, with the null hypothesis that the power-law line fits the data. Since the p-values from the Kolmogorov-Smirnov tests are all not significant, the null hypothesis cannot be rejected and the calculated power-law is an adequate descriptor of the degree distributions for all four networks. C.I., confidence interval. (TIF) [file pcbi.1002029.s001.tif]

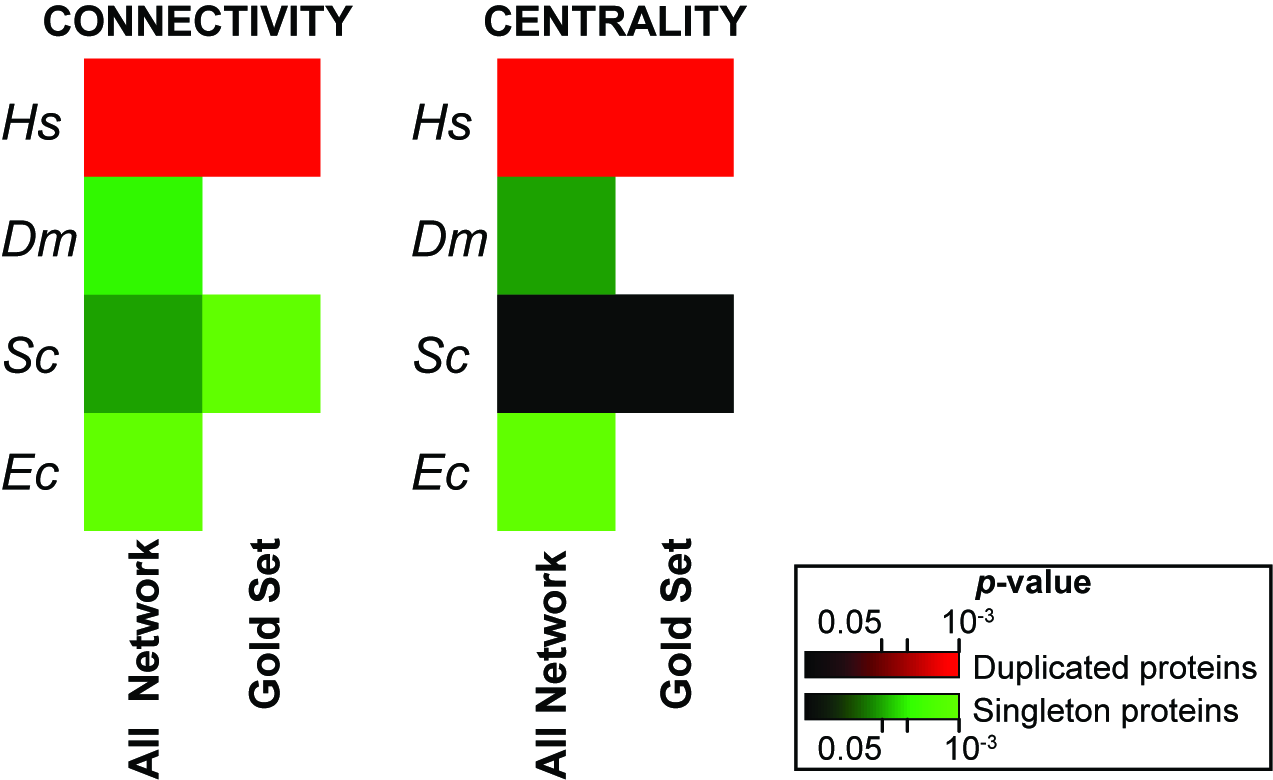

Supplement: Figure S2 — Connectivity and centrality of singleton and duplicated genes in the four networks. Degree and betweenness of proteins encoded by all duplicated and all singleton genes are compared in the four species using the Wilcoxon test. All p-values are transformed into heatmaps where red indicates that duplicated genes encode for significantly more connected or more central proteins than singleton proteins. Green indicates that proteins encoded by singleton genes are significantly more connected or more central than duplicated proteins. Black indicates non-significant p-values. This analysis is done using the entire network for all the four species and the gold set for human and yeast. (TIF) [file pcbi.1002029.s002.tif]

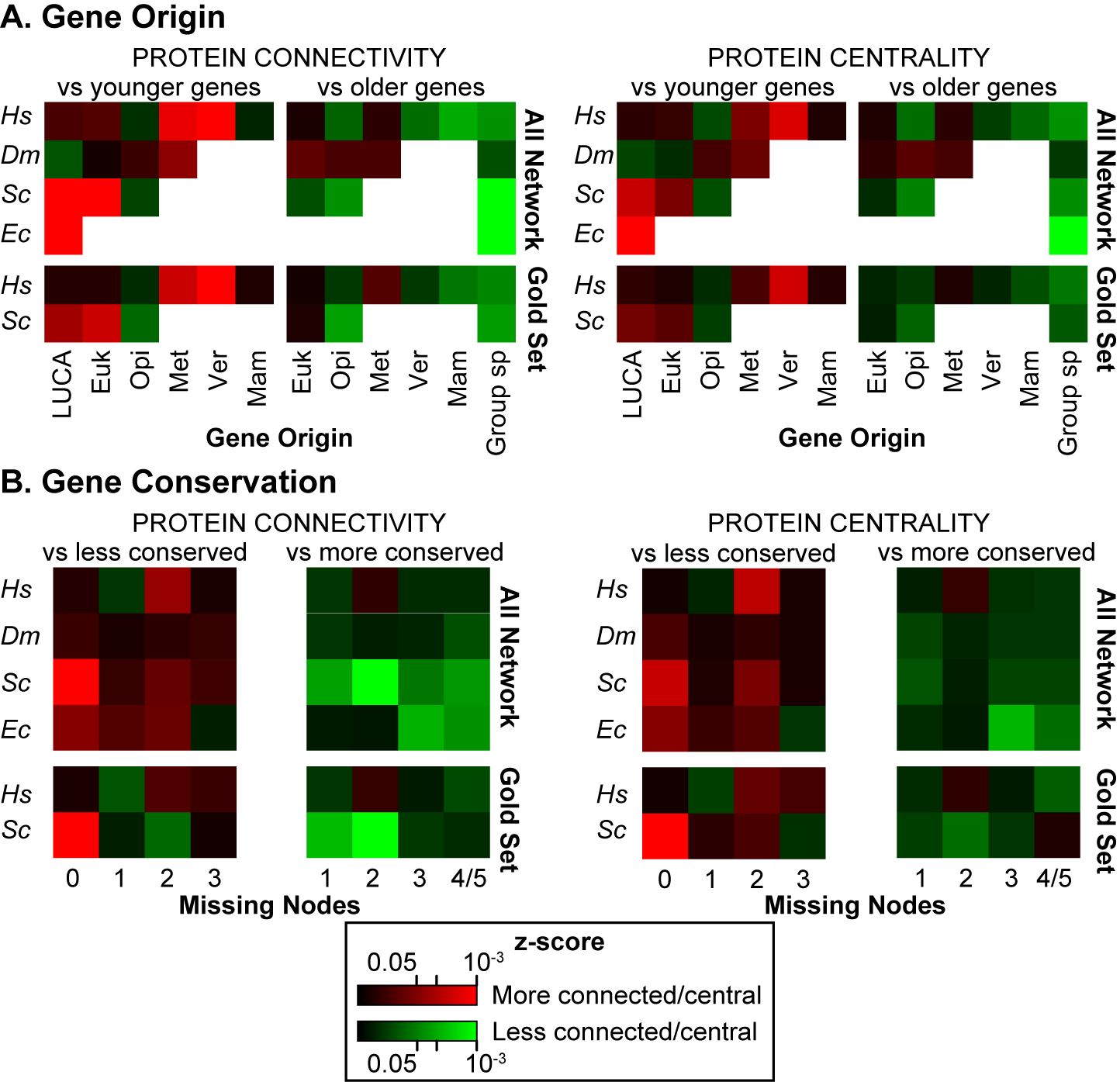

Supplement: Figure S3 — Relationship between gene and network properties measured with randomization tests. Degree (connectivity) and betweenness (centrality) are compared between (A) proteins that originated at a given node and younger or older proteins; and (B) proteins with a given conservation and less or more conserved proteins. In each species, we pick subsets of 500 random genes with a given origin, determine the mean degree and betweenness of the corresponding proteins and compute the difference with 500 younger and 500 older proteins. We repeat the same procedure 100,000 times and derive a z-score as the fraction of randomizations with a negative difference when comparing with younger proteins, and with a positive difference when comparing with older proteins. The same analysis is done for conservations. Each square in the heatmap represents genes that originated at a given internal node or with a given level of conservation. The color represents the z-score. Red is associated with more connected or more central proteins, green is associated with less connected or less central proteins. The lower bound of z-scores is set equal to 10−3. (TIF) [file pcbi.1002029.s003.tif]

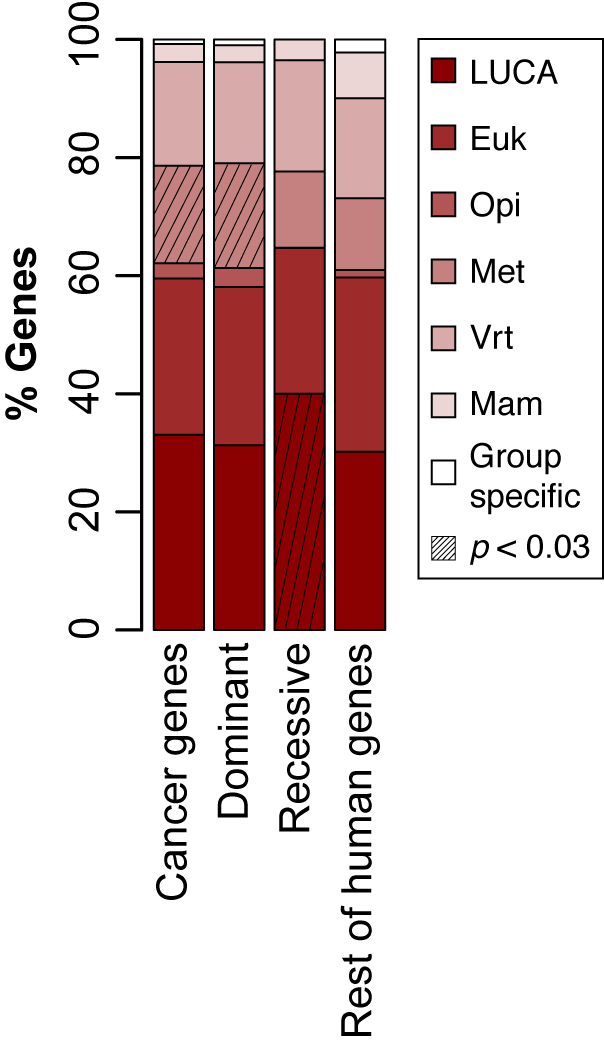

Supplement: Figure S4 — Time of appearance of recessive and dominant cancer genes. The percentage of genes that originated at each of the seven internal nodes of the tree of life is compared between cancer genes and the rest of human genes. The 415 cancer genes are derived from the cancer gene census (frozen at January 11th 2010), and are defined as genes that are causally implicated in tumorigenesis [71]. For 393 of those, the origin can be traced (Table 1), and 310 genes are defined as dominant, and 85 as recessive. Two genes (CBL and PKRAR1A) are included in both lists because they can behave as dominant and recessive. Differences between the appearance of cancer genes and the rest of human genes are calculated using Fisher's exact test and, where significant, are depicted as diagonal lines. (TIF) [file pcbi.1002029.s004.tif]
